# Pantanal virophage: a new virophage species associated with a moumouvirus and a transpoviron, expanding the genus *Sputnikvirus*

**DOI:** 10.1128/jvi.01930-25

**Published:** 2026-04-02

**Authors:** Bruna Luiza de Azevedo, Nidia Esther Colquehuanca Arias, Talita Bastos Machado, João Pessoa Araújo Júnior, Ana Karoline da Nóbrega Nunes Alves, Luiz Carlos Junior Alcantara, Frank O. Aylward, Luiz Henrique Rosa, Rodrigo Araújo Lima Rodrigues, Jônatas Santos Abrahão

**Affiliations:** 1 Laboratório de Vírus, Departamento de Microbiologia, Universidade Federal de Minas Gerais (UFMG) 28114, Belo Horizonte, Brazil; 2 Laboratório de Virologia, Departamento de Microbiologia e Imunologia, Universidade Estadual Paulista (Unesp) 28108, Botucatu, São Paulo, Brazil; 3 Instituto Rene Rachou, Fundação Oswaldo Cruz and Expanded Navigation for Intensive and Optimized Surveillance (NAVIO) Network, Belo Horizonte, Brazil; 4 Department of Biological Sciences, Virginia Tech 1757, Blacksburg, Virginia, USA; 5 Center for Emerging, Zoonotic, and Infectious Disease, Virginia Tech 1757, Blacksburg, Virginia, USA; 6 Laboratório de Microbiologia Polar e Conexões Tropicais, Departamento de Microbiologia, Universidade Federal de Minas Gerais 28114, Belo Horizonte, Brazil; Michigan State University, East Lansing, Michigan, USA

**Keywords:** virophage, Pantanal, moumouviruses, transpoviron, species, taxonomy, *Sputnikvirus*

## Abstract

Amoeba-infecting giant viruses, such as mimiviruses, challenged paradigms of virology since their discovery. Their extensive and complex genomes and particle structures can interact with virophages and transpovirons, revealing unique aspects of the viral ecology and evolution. Here, we describe the isolation of Pantanal virophage, a new virophage obtained from Brazilian pantanal biome. The virophage was found in association with moumouvirus pantanense and a transpoviron sequence. Sequencing and genomic analysis revealed that Pantanal virophage presents a 17,964 bp genome with 21 coding sequences, including two ORFans that code for proteins with completely unknown functions. Phylogeny showed that Pantanal virophage composes a divergent branch within virophages from *Sputnikvirus* genus. Average nucleotide and amino acid identity analysis comparing different virophage sequences indicated that Pantanal virophage could represent a new *Sputnikvirus* species. These findings shed light on the giant virus-virophage-transpoviron triplet diversity, evolution, and ecology, contributing to a future update in virophages taxonomy.

**IMPORTANCE:**

Studies on prospecting, isolation, and characterization of new amoeba viruses are important to provide new information about biology, diversity, evolution, ecology, and taxonomy of these viruses. This work reinforces this importance since we describe Pantanal virophage, a new species of Sputnikvirus found in association with a moumouvirus and a transpoviron. The characterization of Pantanal virophage provided new data and observations regarding the phylogeny and taxonomy of *Sputnikvirus* genus evidencing the need for constant updates in taxonomic classification. This work shows that the efforts for isolation of new amoeba viruses and their characterization can contribute to enriching the knowledge about taxonomy and evolutionary dynamics of these viruses and of their parasitic-associated elements.

## INTRODUCTION

Giant viruses of amoeba have surprised the scientific community since the description of their first representative, the mimivirus, in 2003 (1). With particles that exceed the dimensions (750 nm) of some bacterial cells and linear double-stranded DNA genomes with more than 1.2 million base pairs, mimiviruses represent a milestone in the history of modern virology, and they opened the door to the discovery of new amoeba-infecting viruses. In the last years, it was revealed the great diversity of the *Mimiviridae*. Currently, the family *Mimiviridae* is composed of different subfamilies including the *Megamimivirinae*, which includes genera such as *Mimivirus*, *Moumouvirus*, *Megavirus, Cotonvirus,* and *Tupanvirus* (2). Considering the high structural and genomic complexity of the megamimiviruses, subsequent studies have shown that they have atypical relationships with other organisms, including the possibility of being parasitized by satellite-like viruses, known as virophages (3). These entities parasitize the viral factories of mimiviruses and are only able to form their progeny in the amoeba cytoplasm during co-infection with mimiviruses.

Different virophages have been described in recent years, capable of parasitizing not only mimiviruses but also megaviruses, moumouviruses, tupanviruses, and Cafeteria roenbergensis virus (4–7). The impact of virophage parasitism on the morphogenesis of giant viruses is variable. Some virophages strongly affect the formation of their associated giant virus progeny, while others appear not to interfere with their development (3, 4, 8, 9). The interaction between virophages and their viral hosts is complex and can also be related to specific genetic mobile elements known as transpovirons (10–12). Transpovirons are episomal and linear sequences with around 7 kb that code for their own proteins. It is proposed that transpovirons use the DNA replication machinery of the giant virus and that they can use the virophage to disseminate and to increase their gene expression without affecting the productivity and the gene expression patterns from the virophage or from the giant virus (11, 13).

Besides the virophage isolates, several virophage sequences have been detected through metagenomics, including high-quality metagenome-assembled genomes (MAGs), revealing great diversity of these viruses (14–18). As a result, a recent taxonomic proposal submitted to the ICTV established a restructuring in the taxonomy and nomenclature of virophage-related taxa. In this context, species related to Sputnik- and Zamilon-type virophages were allocated to the family *Sputniviroviridae*, genus *Sputnikvirus*, with two species: *Sputnikvirus mimiviri* and *Sputnikvirus zamilonense*, respectively (2). Because some virophages were isolated, they have enabled fundamental studies into the nature of the virophage–giant virus relationship and allowed for structural analyses of the particles.

Here, we present the discovery of a new virophage of the genus *Sputnikvirus*, named Pantanal virophage. This virus was isolated in association with a moumouvirus and a transpoviron, suggesting a complex interplay among multiple entities within the same host. The Pantanal virophage exhibits intermediate genomic features that position it between previously described virophages of the Sputnik and Zamilon types. These findings not only highlight the continuum of diversity within the *Sputnikvirus* genus but also provide further evidence of the evolutionary plasticity and ecological versatility of virophages in amoebal environments.

## RESULTS

### A moumouvirus-virophage-transpoviron tripartite association obtained from Brazilian Pantanal samples

**Fig 1:**
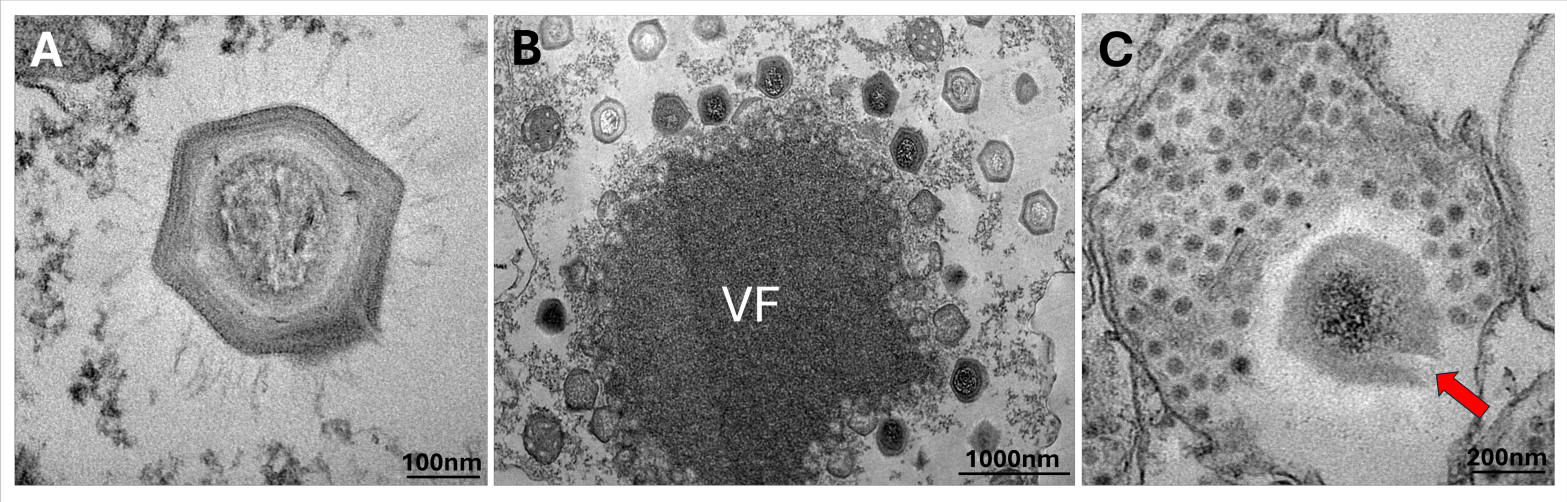
Morphological features of the viral isolates analyzed through transmission electron microscopy (TEM). (**A**) A mimivirus-like particle with a fibril distribution pattern that corresponds to moumouviruses’ pattern. (**B**) The moumouvirus viral factory (VF) in the cytoplasm of *A. castellanii* infected cell, surrounded by newly assembled particles. (**C**) Virophage-like particles inside a vesicle. A putative moumouvirus defective particle (red arrow) is observed inside vesicles (membrane surrounding the moumouvirus and virophage particles).

From the samples collected from Pantanal biome in Brazil, we isolated an amoeba-infecting giant virus using *Acanthamoeba castellanii* as host platform. The isolation was confirmed through transmission electron microscopy (TEM) images that showed the presence of mimivirus-like particles with a scarce amount of surface fibrils attached to the multi-layered capsid (Fig. 1A). The isolated virus exhibits replication cycle characteristics corresponding to those observed for mimiviruses, such as a large electron-dense viral factory, from where we can observe viral particles in formation, budding from the edges of the structure and spreading into the host cytoplasm (Fig. 1B). Also, we observed the presence of smaller particles that were suggestive to be virophage particles (Fig. 1C). These smaller particles were often found inside vesicles, probably exosomes, in the final stages of the giant virus replication cycle. In Fig. 1C, it is possible to observe a putative mimivirus-like defective particle (red arrow) inside the same vesicle where virophage particles are observed.

**Fig 2:**
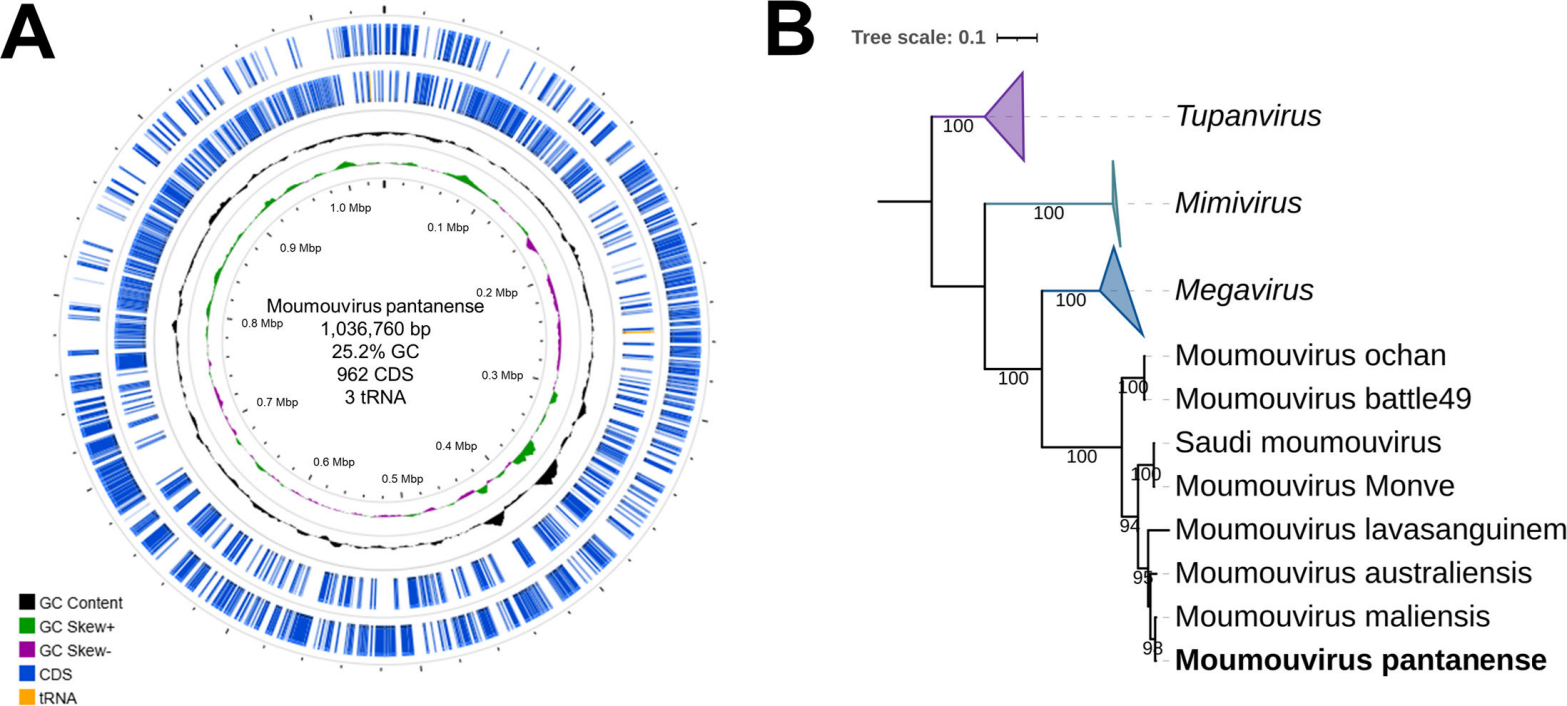
Genomic features and phylogenetic position of Moumouvirus pantanense. (**A**) Genomic map of Moumouvirus pantanense indicating the positions of CDS in blue and tRNA in yellow (forward and reverse strands). Internal rings indicate the GC content and skew. (**B**) Phylogenetic tree based on the alignment of amino acid sequences of DNA polymerase B family including representatives of the family *Mimiviridae*. The tree was rooted using tupanviruses as outgroup. Moumouvirus pantanense is labeled in bold, included in the moumouvirus clade. Bootstrap values above 90 are shown. The tree was built with statistical support based on 1,000 replicates (bootstrap) using the JTTDCMut+F+I+G4 evolutionary model, as selected by ModelFinder. The tree scale bar indicates the number of amino acid substitutions per site.

After sequencing and genome assembly, scaffolds representing the giant virus genome were obtained. Compared to the database (GenBank), these scaffolds had as “best hit” the moumouvirus maliensis genome (>90% of nucleotide identity) and were organized using this sequence as reference resulting in a final genome of 1,036,760 bp with 25.2% GC-content (sequencing coverage: 50×) (Fig. 2A). A total of 962 CDS were predicted, including all the *Nucleocytoviricota* core genes, and only three tRNA genes, including tRNA-His(gtg), tRNA-Cys(gca), and tRNA-Leu(caa). Phylogenetic reconstruction based on DNA polymerase B family gene included the new isolate among moumouviruses, thus confirming the classification of it as a new member of the family *Mimiviridae*, the subfamily *Megamimivirinae* and genus *Moumouvirus* (Fig. 2B). Then, the new isolate was called moumouvirus pantanense. An additional parameter that corroborates with the genomic identification of the isolate as a moumouvirus is its pattern of fibril organization in the particle. Moumouviruses are known to have fewer fibrils that are not uniformly distributed (19), such as is observed in Fig. 1A.

**Fig 3:**
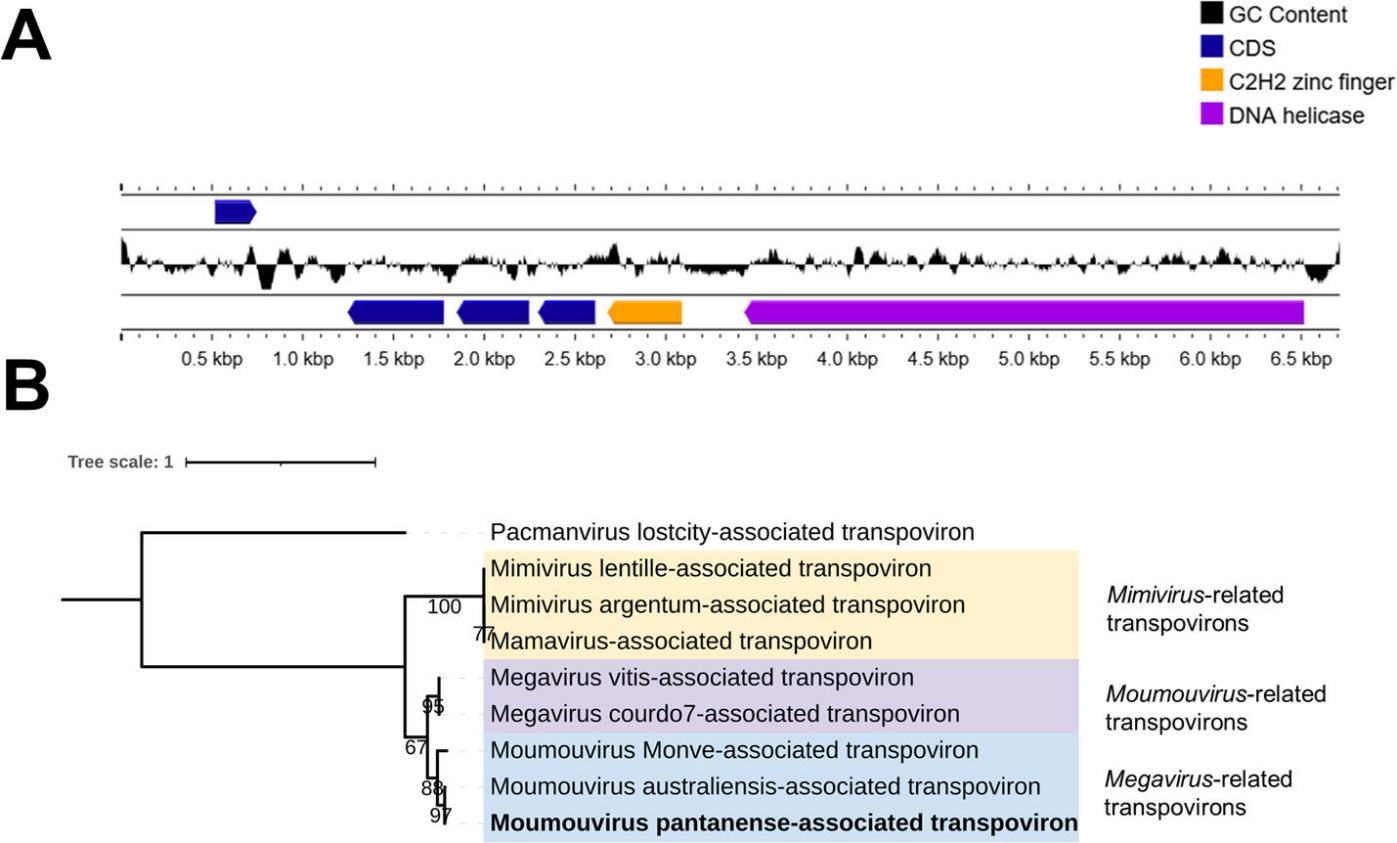
Genomic and phylogenetic characteristics of the Moumouvirus pantanense-associated transpoviron. (**A**) A genome map showing its linear topology and the six encoded ORFs. ORF 6 codes for DNA helicase protein, which is conserved among the known transpovirons. (**B**) Maximum likelihood phylogenetic tree using the DNA helicase amino acid sequence from known transpovirons sequences. The Megamimiviruses-associated transpovirons are highlighted by different colors, and the respective genera are indicated. Moumouvirus pantanense-associated transpoviron is highlighted in bold. The tree was built with statistical support based on 1,000 replicates (bootstrap) using the WAG+F+G4 evolutionary model, as selected by ModelFinder. The tree was rooted on the pacman lostcity-associated transpoviron branch as an outgroup. The tree scale bar indicates the number of amino acid substitutions per site.

Also, a scaffold containing 6,711 bp (sequencing coverage: 51×) and 21.74% GC-content was identified having 99.4% of nucleotide identity with a transpoviron associated with moumouviruses. We analyzed the transpoviron sequence, performing gene prediction and functional annotation. The moumouvirus pantanense-associated transpoviron has six ORFs coding for proteins that vary from 75 to 1,028 amino acids (Fig. 3A). ORFs 1 to 4 code for hypothetical proteins whose functions were not defined yet. ORFs 5 codes for C2H2 zinc finger protein and ORF 6 codes for the largest protein, predicted to be a DNA helicase. This enzyme is a conserved protein among the known transpovirons and can be used to phylogenetic analysis. In transpovirons phylogeny, the known DNA helicase protein sequences form three different clades that correspond to the giant viral lineages to which they are related (*Mimivirus* genus, *Moumouvirus* genus, or *Megavirus* genus) (Fig. 3B).

Another scaffold containing 17,964 bp was obtained with a high copy number, indicated by the sequencing coverage of 1,926×. This sequence had 86.12% of nucleotide identity with a sputnik virophage, confirming the isolation of a new virophage in the sample, which we named Pantanal virophage.

### Genomic and phylogenomic characterization of a new virophage recovered from the Brazilian Pantanal biome

**Fig 4:**
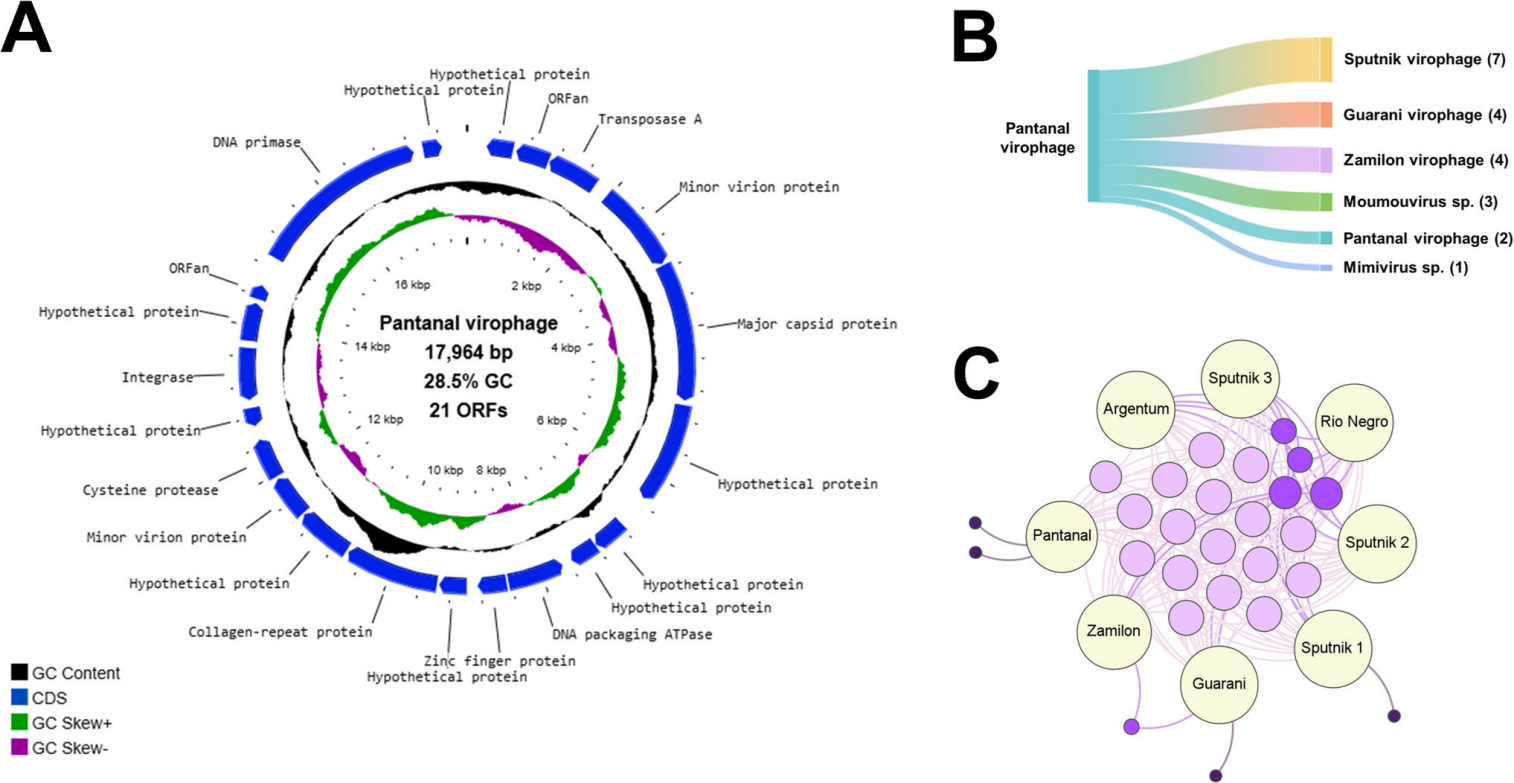
Genomic characterization of Pantanal virophage. (**A**) Genome map showing the circular topology of the Pantanal virophage genome and the distribution of coding sequences (CDSs) throughout the genome. The CDSs, the G-C content, and the G-C skew are illustrated by different ring colors, as indicated in the color legend. To date, minor virion protein is also referred in the literature as minor capsid protein. (**B**) Blastp best hits of Pantanal virophage proteins against NCBI nr database, including different virophages and giant viruses. (**C**) OG-sharing distribution among eight virophage isolates, evidencing the presence of two unique genes of Pantanal virophage. The node sizes are proportional to the connection degree. Light purple nodes represent the core genes, purple nodes are related to satellite genes, and dark purple nodes are the singletons.

In contrast with the moumouvirus and the transpoviron obtained sequences, the Pantanal virophage scaffolds presented a lower nucleotide identity (86.12%) with its best hit from GenBank. Pantanal virophage genome sequence has a GC content of 28.45% and 21 ORFs coding for proteins that vary from 62 to 775 amino acids in size. By searching for similar sequences in databases, we were able to identify conserved proteins among the virophages such as the major capsid protein (MCP), the minor virion protein (mCP), the DNA packaging ATPase, and the cysteine protease (Fig. 4A). When searching for similar sequences in the GenBank database, it is possible to observe that the Pantanal virophage proteins matched with sequences from different virophages. The most part (33.3%, *n* = 7) of Pantanal virophage proteins showed a higher identity with Sputnik virophage 1; four proteins (19%, *n* = 4) had as “best hit” a Guarani virophage and Zamilon protein sequences (Fig. 4B). The remaining proteins had as best hits giant virus sequences (moumouvirus and mimivirus). In addition, Pantanal virophage has two ORFs coding for proteins to which there are no similar sequences in databases, the so called ORFans, which represents 9.5% of its proteome (Fig. 3B). Structural analysis of these ORFans using AlphaFold 3 and Phyre2 resulted in low confidence models that were then discarded. We constructed clusters of orthologous groups of genes (OGs) considering the virophage isolates of the genus *Sputnikvirus*, and as expected, they share most of the genes among each other due to their high genomic similarity (Fig. 4C). A total of 26 OGs were obtained, 17 being shared by all isolates, thus constituting the core-genome of Sputnik-related viruses. Only four genes were considered singletons, two associated with Pantanal virophage (the two predicted ORFans), one with Guarani virophage, and another with the first isolated Sputnik virophage (Fig. 4C).

**Fig 5:**
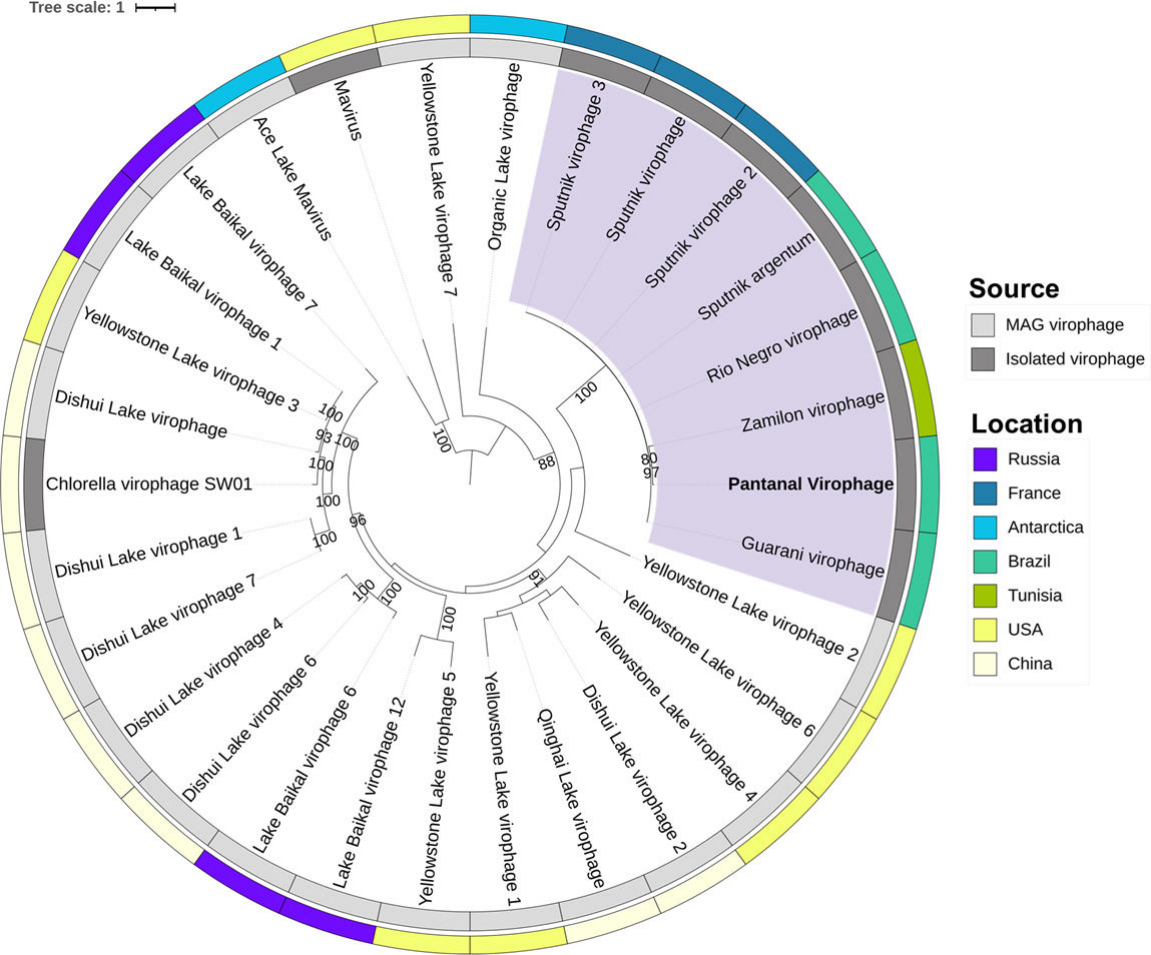
Major capsid protein (MCP) phylogeny based on amino acid sequences from different virophages. Pantanal virophage is labeled in bold. Sequences highlighted in purple are classified within the current genus *Sputnikvirus*. The inner ring indicates the source of the sequences, with isolated virophages in dark gray and MAG-related virophages in light gray. The outer ring indicates the country from where the virophages were isolated or identified with different colors for each country as indicated in the figure. This maximum likelihood phylogenetic tree was built with statistical support based on 1,000 replicates (bootstrap) and was rooted at the midpoint. The best model selected by IQ-TREE (ModelFinder) for the tree was rtREV+F+R3. The scale bar represents the number of amino acid substitutions per site.

**Fig 6:**
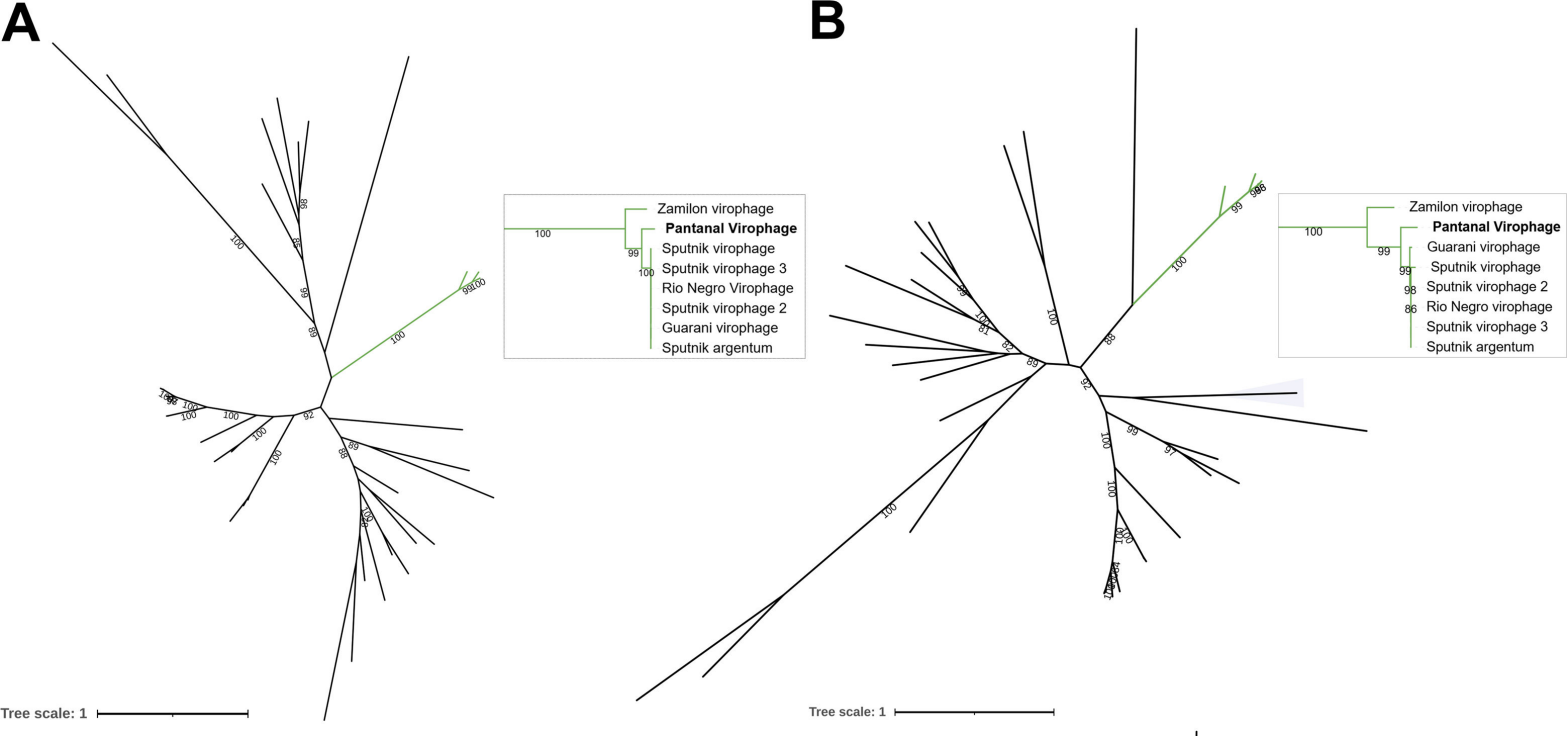
Phylogenetic reconstructions based on virophage conserved genes. (**A**) Unrooted ATPase-based tree. (**B**) Unrooted mCP-based tree. In both trees, the clade representing the genus Sputnikvirus is labeled in green and exhibited in details with the Pantanal Virophage labeled in bold. Only bootstraps higher than 80 are shown, and the scale bars represent the number of amino acid substitutions per site. The best models, selected by IQ-TREE (ModelFinder), were rtREV+F+I+G4 for ATPase tree and Blosum62+F+I+G4 for mCP tree.

**Fig 7:**
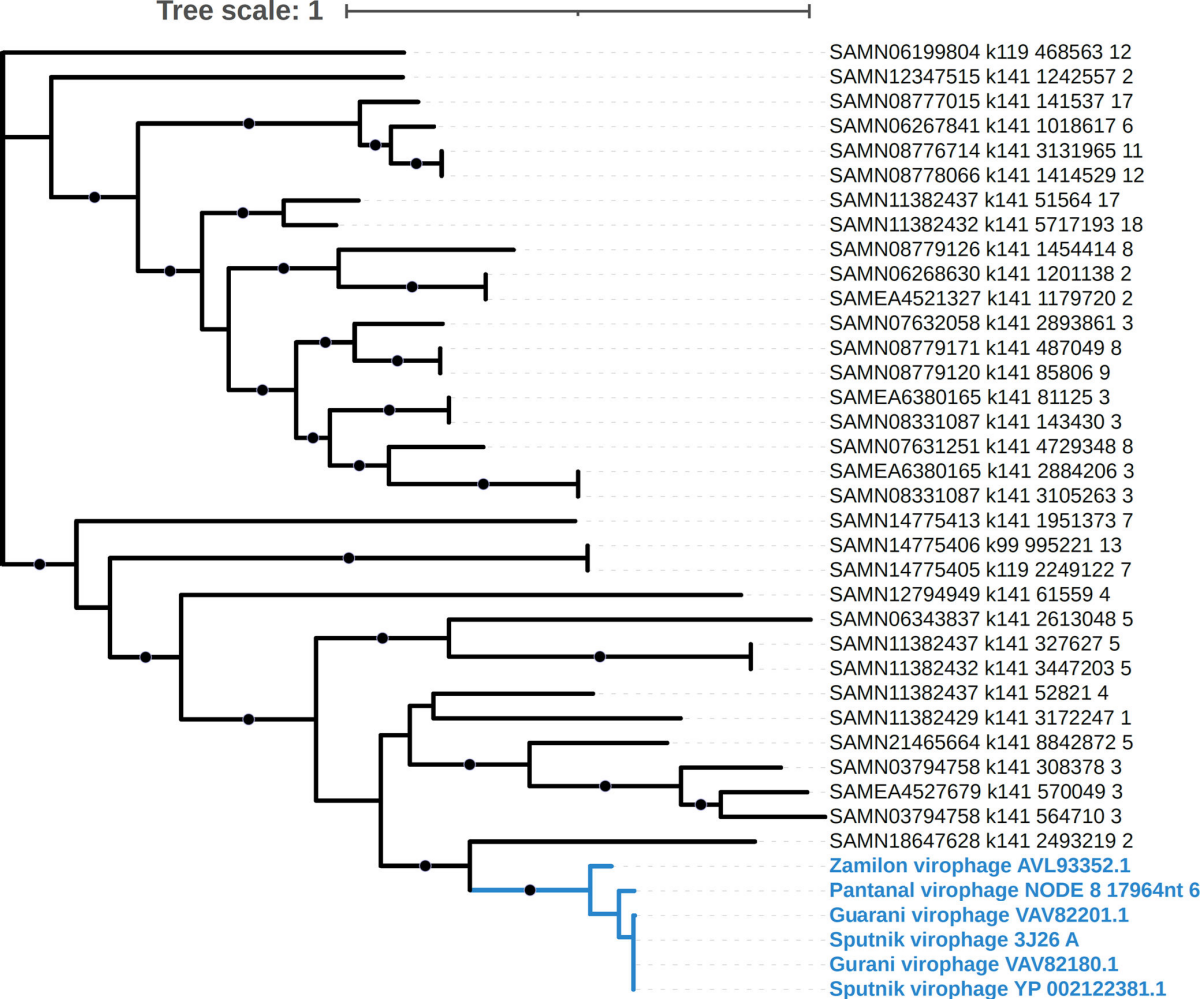
Unrooted phylogenetic tree of the Pantanal virophage major capsid protein (MCP) together with close relatives in NCBI and the best hits recovered from metagenomes. Black circles denote nodes with ultrafast bootstrap values >95. The clade that includes the Pantanal virophage is highlighted in blue and names are provided in bold.

**Fig 8:**
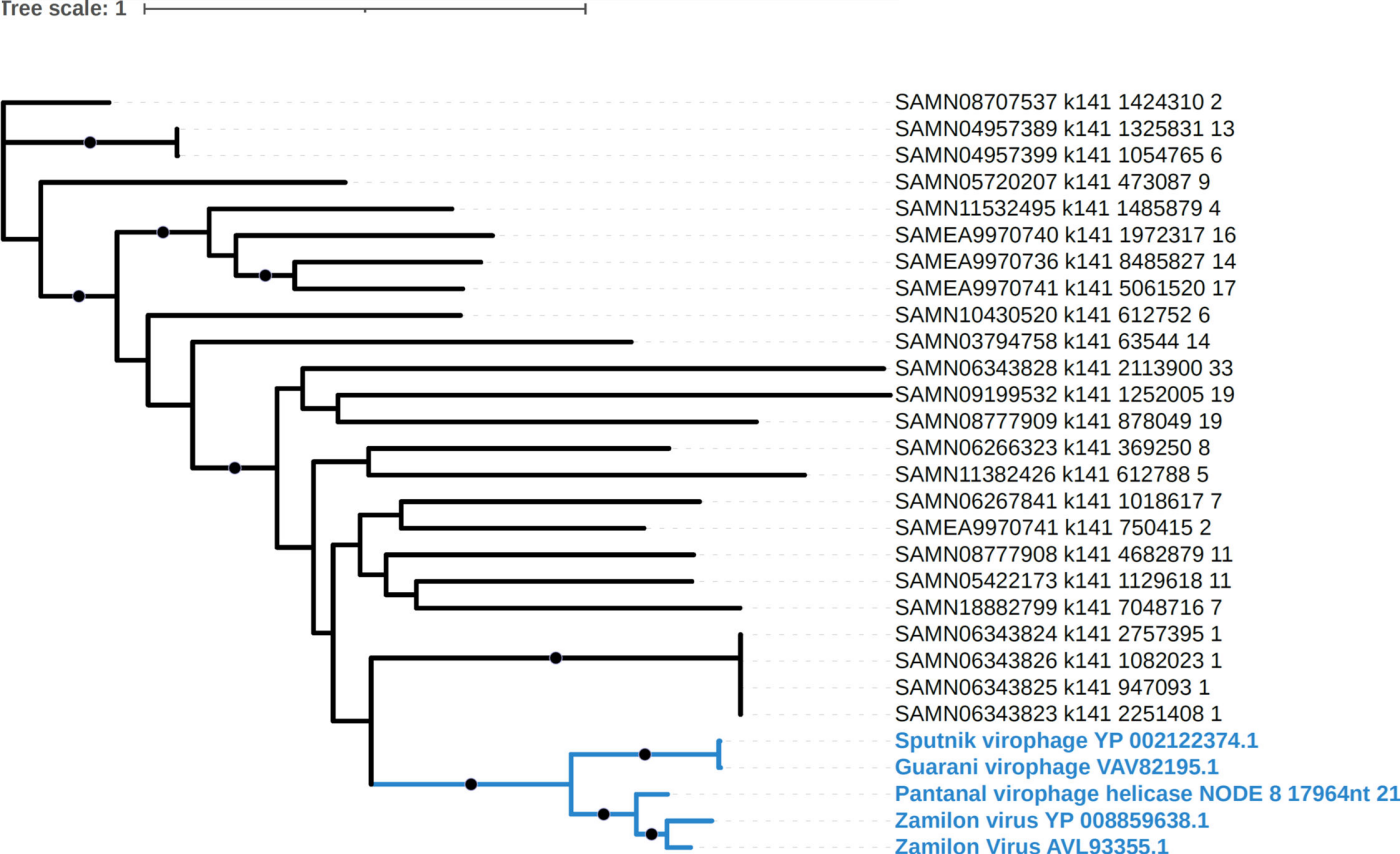
Unrooted phylogenetic tree of the Pantanal virophage helicase protein together with close relatives in NCBI and the best hits recovered from metagenomes. Black circles denote nodes with ultrafast bootstrap values >95. The clade that includes the Pantanal virophage is highlighted in blue and names are provided in bold.

To better understand the relationship between Pantanal virophage and the other virophages, we performed a phylogenetic analysis. The MCP phylogeny revealed that Pantanal virophage is included within the Sputnik and zamilon virophage clade, that is, the genus *Sputnikvirus* (Fig. 5). In our analysis, we included both isolated and MAG-related virophages since most of the known virophage sequences derived from non-isolated viruses. Sequences included in this analysis were recovered from different regions around the world. Notably, most isolated virophages were recovered from Brazilian samples, followed by French samples and only one isolate to date obtained from samples collected at USA and China (Fig. 4). Also, it is possible to observe that Pantanal Virophage forms a divergent branch within the virophages belonging to *Sputnikvirus* genus, considering phylogenetic reconstruction using other conserved genes, including ATPase (Fig. 6A) and mCP (Fig. 6B). Considering that few virophages have been isolated so far, we performed new phylogenetic analyses using the Major Capsid Protein (Fig. 7) and Helicase (Fig. 8) genes, combined with a search for virophage sequences across more than 16,000 metagenomic databases. This analysis allowed the incorporation of additional sequences into the phylogeny and ultimately confirmed the existence of the branch containing the isolated virophages, while reaffirming the position of the Pantanal virophage as an independent branch within the group.

**Fig 9:**
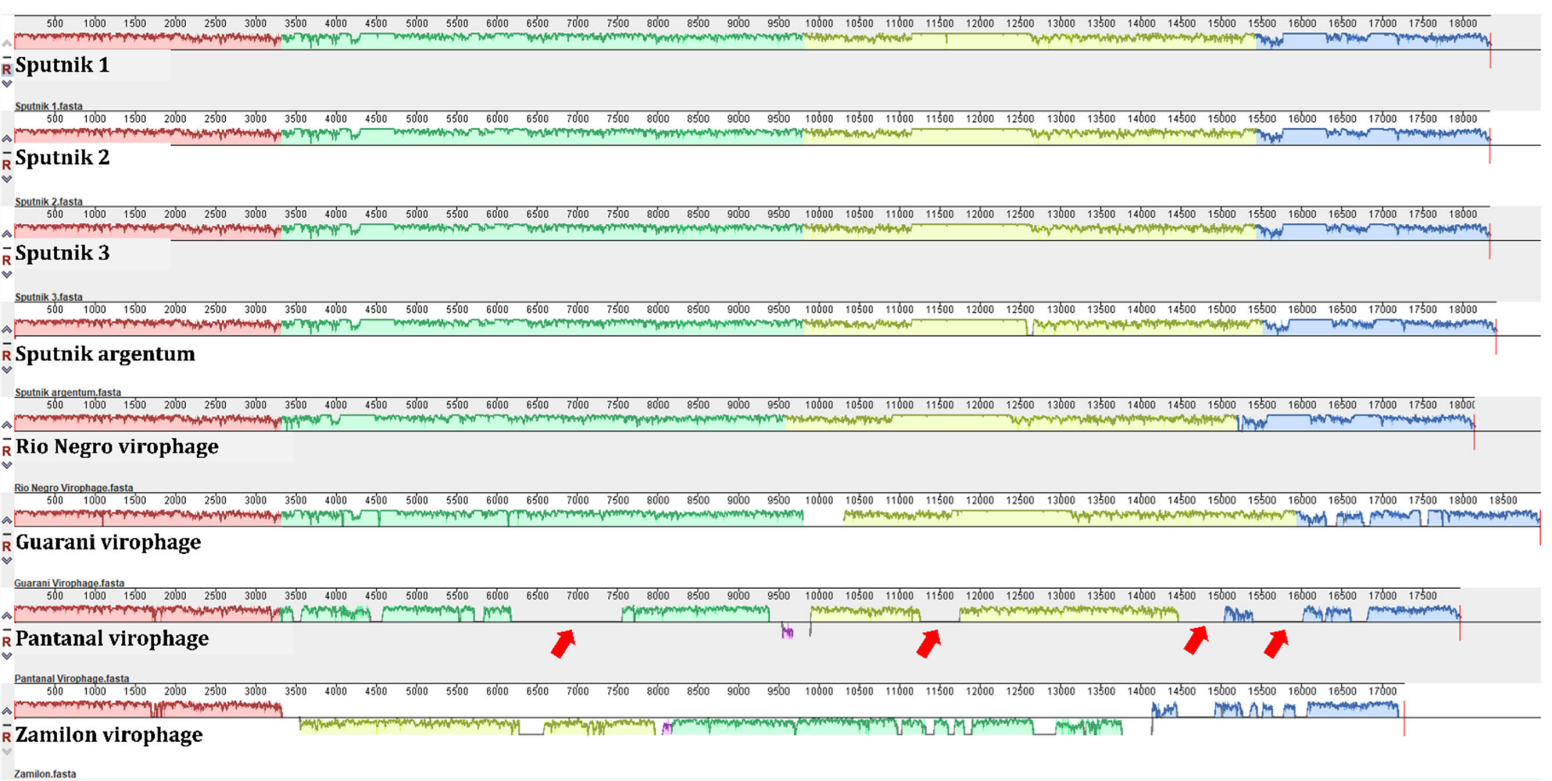
Genomic synteny based on complete sequences from different Sputnikvirus. Each line represents a different virophage sequence. The sequence identification is found below each line. In this analysis, the blocks of the same color indicate similar regions between sequences. The areas without any colored blocks represent regions exclusive to that virus, that is, which do not show similarity with the other viruses used in the analysis. Some of the exclusive regions from Pantanal virophage are indicated by red arrows. Note: All the genomic sequences were adjusted to start from the major capsid protein (MCP) aiming to facilitate interpretation of this figure because they have a circular topology.

Additionally, we evaluated the genome synteny of members of the genus *Sputnikvirus* and the Pantanal virophage. In general, the Sputnikvirus virophages have well-conserved synteny, which was expected due to the high genomic identity among them. Nevertheless, we observed that the Pantanal virophage has at least four genomic regions that do not show similarity with the other viruses used in the analysis (Fig. 9, red arrows). These data corroborate with phylogenies that show Pantanal virophage as a divergent sequence in this group.

### Nucleotide and amino acid identity analyses reveal Pantanal virophage as a new virophage species

**Fig 10:**
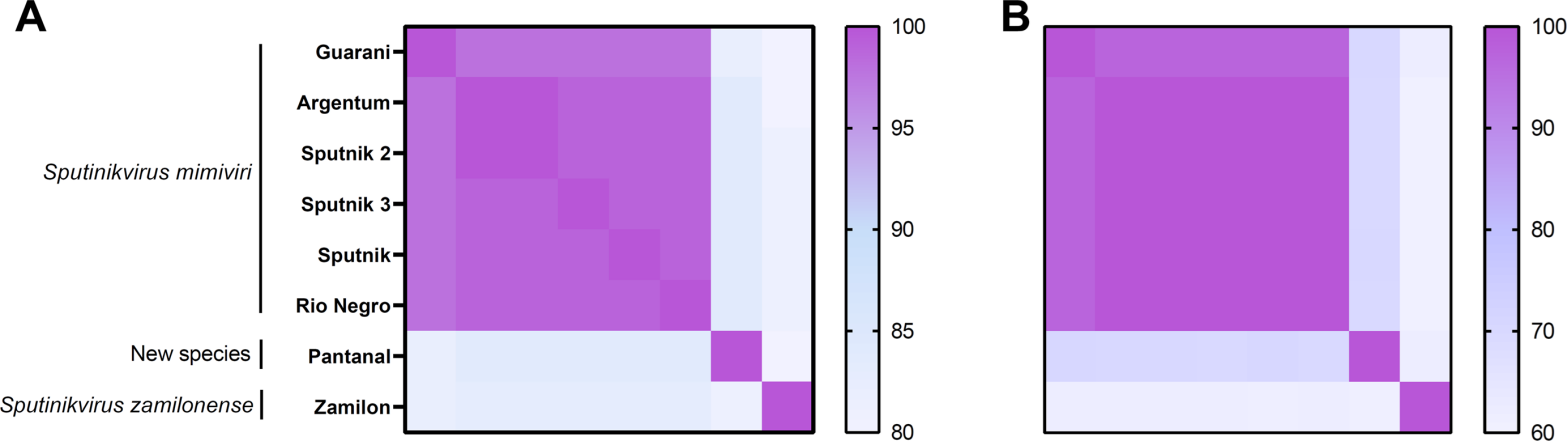
Average nucleotide identity (ANI) and average amino acid identity (AAI) analyses comparing different virophage sequences from genus *Sputnikvirus*, including Pantanal virophage. The analysis is based on a similarity matrix composed of (**A**) ANI and (**B**) AAI values. The two current species from the genus (*Sputnikvirus mimiviri* and *Sputnikvirus zamilonense*) are indicated. Pantanal virophage is indicated as a new species. ANI values range from 80% to 100%, while AAI values range from 60% to 100%.

The sequences of *Sputnikvirus* genus isolates were submitted to Average Nucleotide Identity (ANI) and Average Amino Acid Identity (AAI) analyses. In both ANI and AAI analysis, it is possible to delineate three major groups within the genus *Sputnikvirus* based on a cutoff of ANI and AAI >95% (Fig. 10). This ANI cutoff was previously considered a parameter to define viral species for other virophages (20). The first group represents *Sputnikvirus mimiviri* species and its isolates with ANI values that vary from 98% to 100% and AAI values ranging from 97.3% to 100% (Fig. 10). Guarani virophage is the sequence with a smaller ANI and AAI values when compared with the other isolates from *Sputnik mimiviri* species. The second group represents *Sputnikvirus zamilonense* species. The results show ANI < 83% and AAI < 62% corroborating with its classification as a different species (Fig. 10). The third group corresponds to Pantanal virophage sequence. When compared to the other sequences, the new isolate has ANI values ranging from 80% to 84% and AAI values ranging from 61.8% to 70.7%. These data corroborate with our genomic and phylogenetic analyses, revealing Pantanal virophage as new species within the genus *Sputnikvirus*.

## DISCUSSION

The findings and analysis described here represent the isolation of a tripartite system involving moumouvirus pantanense, its associated transpoviron, and the Pantanal virophage. This kind of relationship was already described before for members of the genus *Mimivirus* (10, 12) and genus *Megavirus* (4, 11). For genus *Moumouvirus,* it was described the detection of associated transpovirons sequences, but not simultaneously with virophages (10, 11). Also, it was described that zamilon virophage is able to infect moumouviruses, but they were not isolated from the same sample (4). This work describes, to our knowledge, the first report of simultaneous isolation of this tripartite system from the same sample involving moumouviruses. Also, it is the first report of this interaction in Pantanal biome which adds new information to the ecology and diversity of giant viruses and virophages.

The isolation of “Moumouvirus pantanense” was confirmed both morphologically and genomically. The particle has fewer fibrils with a non-uniform distribution corroborating with the fibril pattern described previously for moumouviruses (19). Genomic comparisons further support this classification, with >90% nucleotide identity and phylogenetic relationship with other moumouvirus isolates, placing the virus within the genus *Moumouvirus*. The identification of a closely related transpoviron, sharing >99% identity with known Moumouvirus-associated transpovirons and the phylogeny based on the conserved helicase gene of the transpoviron reinforces the established three-lineage model corresponding to *Mimivirus*, *Moumouvirus*, and *Megavirus* genera, supporting the hypothesis that transpovirons are co-evolving with their associated viral lineages (11, 12).

The major contribution of this study is the characterization of the Pantanal virophage. The description of two ORFans may reflect adaptation to a unique ecological niche or host interaction pattern, considering the different location where it was isolated and the tripartite system with which the virophage is related. These unknown proteins could play key roles in the biology of the virophage which could warrant the need for an in-depth functional characterization. Also, the presence of protein-coding genes showing highest similarity to multiple virophage (including Sputnik, Guarani, and Zamilon) and giant viruses lineages deserves future investigation, as it may be related to mosaicism of Pantanal virophage genome, as it is observed for virophages in general (3). This kind of genetic mosaicism observed in virophages, in general, can be associated with horizontal gene transfer possibly facilitated by co-infection scenarios due to the amoebas phagocytic behavior or/and to the presence of genetic mobile elements like the transpoviron (10, 21–23).

Although it clusters within the *Sputnikvirus* genus, Pantanal virophage exhibits several features that distinguish it from previously described members. The relatively low nucleotide identity (86.12%) with its closest relative, along with divergent phylogenetic placement and unique genomic regions lacking synteny with other Sputnikvirus genomes, suggests that Pantanal virophage constitutes a new group of virophages. These findings are reinforced by ANI and AAI analyses, which show that Pantanal virophage falls below the 95% identity threshold previously used to demarcate viral species (20), suggesting that it can be classified as a new species within the *Sputnikvirus* genus.

Taking together, our results extend the known biodiversity of virophages and their associated elements and underscore the importance of further exploring underrepresented ecosystems for novel giant viruses’ discovery. The identification of Pantanal virophage as a new species within genus *Sputnikvirus* not only expands the known phylogenetic and taxonomic diversity of this group but also raises important questions about virophage-host coevolution, adaptation, and ecological impact in the ecosystems.

## MATERIALS AND METHODS

### Viral isolation

The sample was collected from sewage samples obtained during NAVIO (Expanded Navigation for Intensive and Optimized Surveillance) expedition in Porto Murtinho city (21°41'36"S 57°53'25"W), Mato Grosso do Sul State, Brazil. This city is located at the southern end of the Pantanal biome, which is a South American ecosystem known for its wetlands and great biodiversity. The giant virus and the virophage were isolated following a well-established protocol based on the inoculation of the collected samples on 96-well plates containing *Acanthamoeba castellanii* cells (24). Non-infected wells were reserved in the same conditions to be used as the experiment control. The inoculated wells were often observed aiming to search for cytopathic effects (CPE), such as rounding cells and cellular lysis. When the CPE was observed, the well content was collected and analyzed through transmission electron microscopy (TEM) aiming to confirm the isolation.

### Viral production and titration

To conduct genomic analysis, we needed to produce a great quantity of the isolated virus. Then, cell culture flasks filled with 1.4 × 10^7^ *Acanthamoeba castellanii* cells and 35 mL of peptone-yeast extract-glucose (PYG) medium, supplemented with penicillin (100 U/mL; Cellofarm, Brazil), streptomycin (100 μg/mL; Sigma-Aldrich, USA), and amphotericin B (0.25 μg/mL; Cultilab, Brazil) were inoculated with the isolates considering at a multiplicity of infection (MOI) of 0.01 for the giant virus as performed before (25). The viral titration was obtained and calculated using the end-point method (26). The infected cells were incubated at 30°C. Non-infected cells maintained in the same conditions were used as control. When viral-induced CPE was observed, the flask’s content was collected, and then it was ultracentrifuged (36,000 × *g*) in tubes containing a 22% sucrose cushion for 30 min. The purified viral particles were stored at −20°C in microtubes containing 300 μL of phosphate-buffered saline (PBS 1×).

### Electron microscopy analysis

The samples containing the isolated viruses were processed to TEM analysis. A total of 7 × 10⁶ *Acanthamoeba castellanii* cells, grown in a cell culture flask containing 25 mL of PYG medium, were infected with the virus at a multiplicity of infection (MOI) of 0.01. After the observation of CPEs, the flask’s content was washed twice using 0.1 M sodium phosphate buffer. After, the sample was fixed in a solution containing glutaraldehyde (2.5%) and sodium phosphate buffer (0.1 M) for 2 h. Post-fixation methodology was performed by using 2% osmium tetroxide followed by the inclusion of the sample in Epon resin to allow the sectioning. A Spirit Biotwin FEI transmission electron microscope (120 kV) was used to analyze the samples at the Microscopy Center of the Federal University of Minas Gerais (CM-UFMG).

### Genome sequencing, assembly, and annotation

The virus-virophage-transpoviron sequences were obtained using an Illumina MiSeq instrument with a single-end library using the Illumina DNA Prep Kit (Illumina Inc., San Diego, CA, USA). The obtained reads were submitted to FastQC program to a sequence quality control. The reads were trimmed using the Trimmomatic tool and filtered to retain sequences with Phred values higher than 28 (27). The sequences were assembled *de novo* using Spades 3.12 program with default parameters (28, 29). After assembling, the scaffolds were compared with the NCBI database, using BLASTn against NCBI non-redundant nucleotide database (nr/nt) with the *e*-value threshold of 10^−3^. FastQC, Trimmomatic, and Spades were all used in this work through the Galaxy server (30). Virophage and transpoviron genes prediction was performed using the Prodigal software (31) with the meta mode parameter. The functional annotation of the proteins was obtained through BLASTp (*e*-value threshold: 10^−3^) analysis against the NCBI non-redundant protein sequence (nr) database.

### Genomic synteny

The comparison between different virophage genomic sequences was performed by making a genome synteny analysis using the MAUVE program version 20150226, with its default parameters (32). The sequences used in this analysis were obtained from the NCBI GenBank database available until February 2025: Sputnik 1 (NC_011132.1); Sputnik 2 (JN603369.1); Sputnik 3 (JN603370.1); Rio Negro virophage (MG676470.1); Sputnik argentum (OL770071.1); Guarani virophage (LS999520.1); Zamilon virophage (NC_022990.1). A manual curation was performed aiming to standardize the sequences to start with the major capsid protein since virophages have genomes with circular topology to allow proper comparison of genome organization.

### Phylogenetic analysis

The sequences for alignment and phylogenetic analysis were obtained through a search for similar sequences against the NCBI non-redundant protein sequences (nr) database using BLASTp with an expected threshold of 10^−3^. The data sets were aligned with the MUSCLE algorithm using the MEGA X software (33, 34). Maximum likelihood phylogenetic trees were obtained using the IQ-TREE software (version 1.6.12) with 1,000 bootstrap replicates as branch support (35). The best-fit substitution models were determined using the ModelFinder algorithm within IQ-TREE (36). The resulting phylogenetic trees were visualized and edited using iToL v6 (37).

### Average nucleotide and amino acid identity analyses

The ANI analysis was performed using whole-genome sequences obtained from NCBI database as described before for genomic synteny. These genomic sequences were submitted to FastANI (38) by using the Galaxy Server (30). Average amino acid identity (AAI) was calculated using the AAI calculator available at http://enve-omics.ce.gatech.edu/aai/. It analyzed reciprocal best hits (two-way AAI) between two virophages protein genomic data sets. A minimum identity cutoff of 20% was considered for AAI. The ANI and AAI obtained data were plotted in heatmaps using GraphPad Prism 9 software.

### Pantanal virophage metagenome search methods

To search for relatives of the pantanal virophage across a range of different habitats, we surveyed 16,801 metagenomes from the SPIRE database (39). We chose the metagenomes in this set such that they represented a wide range of environments, including freshwater ponds and lakes, marine habitats, soil and sediment samples, engineered systems such as wastewater treatment plants, and others. For this analysis, we downloaded the assemblies that were already available on the SPIRE database and considered only contigs >5 kbp in length. We predicted proteins from these contigs using Prodigal v. 2.6.3 (31), and compared the protein predictions to the major capsid protein (MCP) and helicase of the Pantanal virophage using lastal v. 959 (parameters -m 100 -u 2) (40). The best hits for the MCP and helicase were manually inspected. For the MCP, all hits with bit scores >350 were retained, while for the helicase, a score cutoff of 500 was used due to the long length of this protein. We then aligned the best hits for both the MCP and helicase with their homologs encoded in the Sputnik, Zamilon, and Guarani virophages using Muscle v. 5.1 (41), trimmed the alignment using trimAl v1.4.rev15 (parameter -gt 0.1) (42), and constructed a phylogenetic tree using IQ-TREE v. 2.2.2.7 (43) with ultrafast bootstraps (44) (LG+F+R10 model).

### Structural analysis of ORFans

For better understanding of the genes that were considered ORFans, structural analysis was conducted using AlphaFold 3 (45) and Phyre2 (46).

## Data Availability

The sequences generated in this study are publicly available in GenBank under the accession numbers PX975496 to PX975498.
